# Supplementary material for: Adaptogens in Long-Lasting Brain Fatigue: An Insight from Systems Biology and Network Pharmacology
Source: Pharmaceuticals (Basel). 2025 Feb 15;18(2):261. doi: 10.3390/ph18020261 (PMC12128733; doi:10.3390/ph18020261)
Supplement: Supplementary file 1 [file pharmaceuticals-18-00261-s001.zip › pharmaceuticals-3470307-supplementary.pdf]

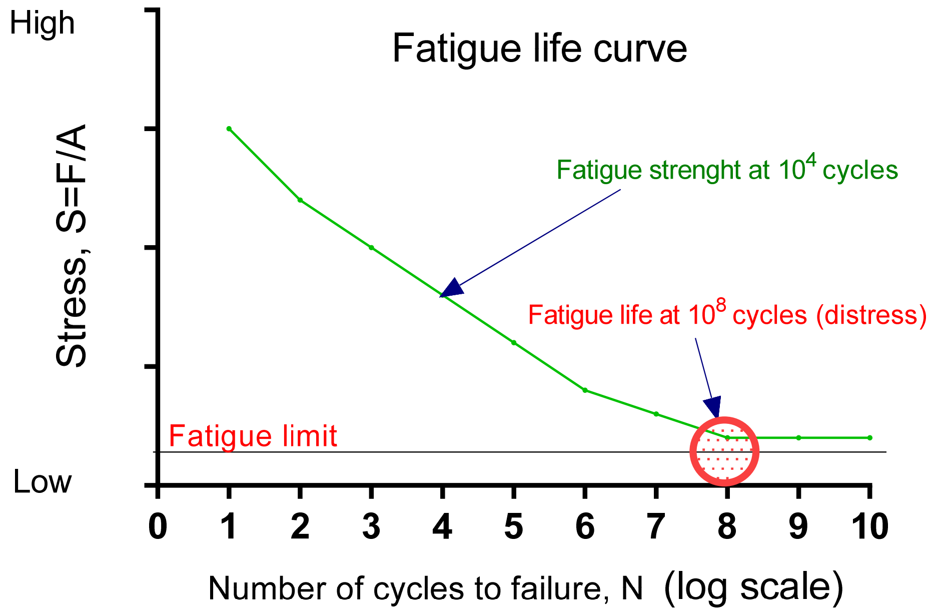

Figure S1. The representative Wöhler's Fatigue Life (S-N) curve shows the number of cycles that cause failure at a specified stress level, as taken from the S–N plot, modified from [72].

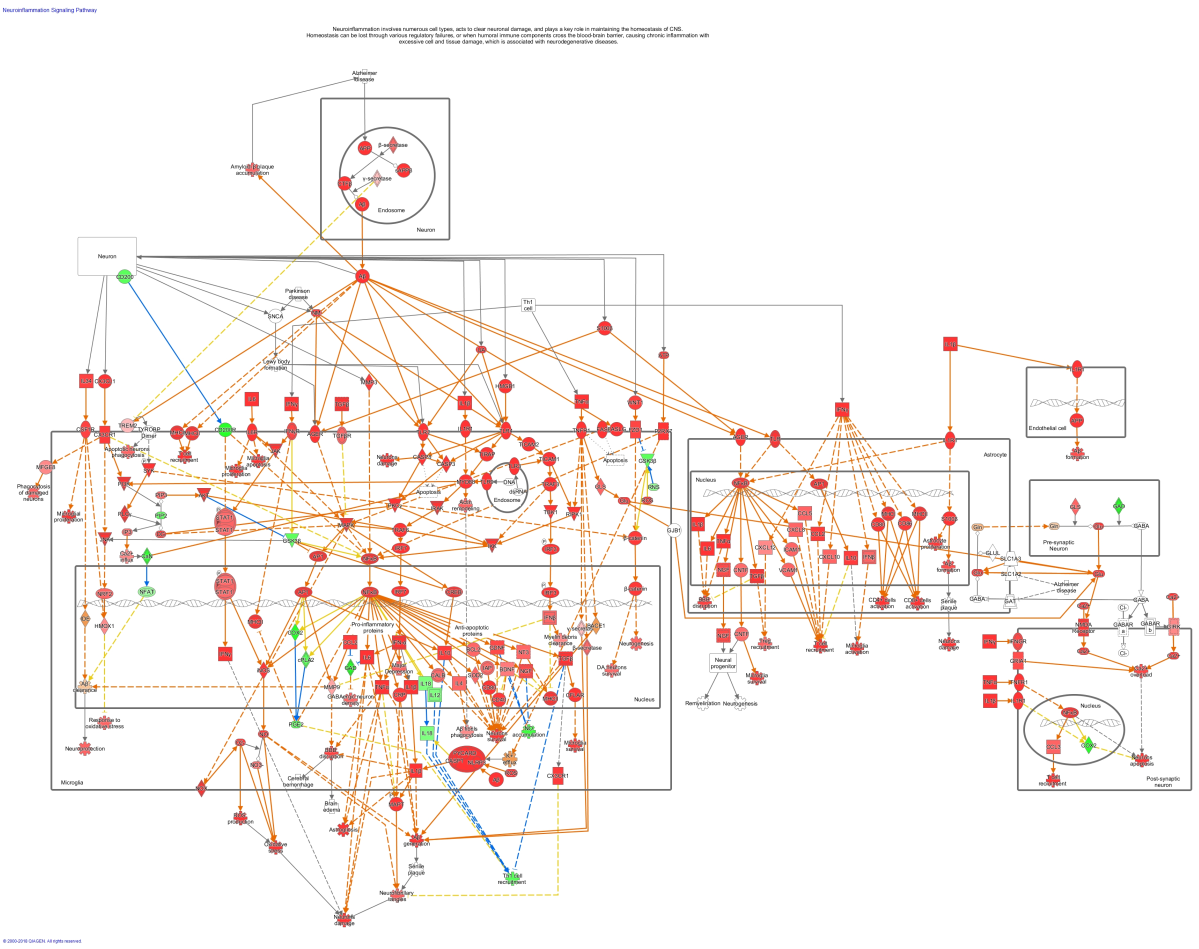

Figure S2. Activation of neuroinflammation signaling pathways in neuroglia cell culture [67].

Table S1. Most essential genes regulated by adaptogens and associated signaling pathways, biological processes, physiological functions, and diseases; adapted from [66,67].

Type(s)	Gene symbol	Protein name	Signaling pathways	Biological processes	Physiol. functions	Diseases
Hormones	*CRH*	Corticotropin-releasing hormone	2	54	7	18
	ACTH	Adrenocorticotrophic hormone; ACTH	2			
	UCN	Urocortin (corticotropin-releasing factor family)	1	53	7	4
	GNRH1	Gonadotropin-releasing hormone 1	3	25	10	12
Transmembrane receptors	*TLR9*	Toll-like receptor 9, member of PI3K (complex)	152	65	7	66
	CHRNE	Cholinergic receptor nicotinic epsilon subunit	3	12	8	22
	PRLR	Prolactin receptor	2	17	7	11
G-protein coupled receptor	*CHRM4*	Cholinergic receptor muscarinic 4	5	11	5	173
Nuclear receptor	*RORA*	RAR-related orphan receptor A	Melatonin signaling	-	16	17
Transcription regulators	*STAT5A*	Signal transducer and activator of transcription 5A	19	57	13	10
	FOS	Fos proto-oncogene, AP-1 transcription factor subunit	21	37	15	52
	FOXO6	Forkhead box O6	3	7	5	10
Kinases	*FLT1*	Fms-related tyrosine kinase 1	9			
	MAPK10 JNK, SAPK1	Mitogen-activated protein kinase 10, c-Jun N-terminal kinase	77	12	11	8
	MAPK13 p38, SAPK2	Mitogen-activated protein kinase 13, p-38 M.A.P. kinase	59	14	10	15
	PRKCH	Protein kinase C eta	72	15	11	20
	PKA	protein kinase A ACTH-induced	cAMP/PKA/CREB signaling			
	PKB	Protein kinase B - AKT				
Metabolic enzymes	*GUCY1A2*	Guanylate cyclase 1 soluble subunit alpha 2	19	4	6	32
	HSPA6	Heat shock protein family A (Hsp70) member 6	6	3	5	3
	PDE3B	Phosphodiesterase 3B	16			
	PDE9A	Phosphodiesterase 9A	6			

Upregulated genes are shown in red, while downregulated genes are in blue.

Table S2. The effects of adaptogens on canonical pathways are commonly involved in regulating adaptive stress response signaling, adapted from Reference [66,67].

**Canonical Pathways**
AMPK signaling
Axonal guidance signaling
Calcium signaling
cAMP-mediated signaling
Cardiac β-adrenergic signaling
Chronic obstructive pulmonary disease signaling
Colorectal cancer metastasis signaling
Corticotropin-releasing hormone signaling
CREB signaling in neurons
CXCR4 signaling
Dendritic cell maturation signaling
Dopamine-DARPP32 feedback in cAMP signaling
eNOS signaling
Glutamate receptor signaling
GP6 signaling pathway
G-protein-coupled receptor signaling
Inositol pyrophosphate biosynthesis
Leptin signaling in obesity
LPS-stimulated MAPK signaling
Melatonin signaling and degradation
Neuroinflammation signaling pathway
Neuropathic pain signaling in dorsal horn neurons
Nitric oxide signaling in the cardiovascular system
NRF2-mediated oxidative stress response signaling
Opioid signaling pathway
Protein kinase A signaling
Relaxin signaling
Renin–angiotensin signaling
Osteoblasts, osteoclasts, and chondrocytes in rheumatoid arthritis signaling
Salvage pathways of pyrimidine nucleotide signaling
Sperm motility signaling
Super pathway of inositol phosphate compounds signaling
Synaptic long-term depression signaling
Telomere extension by telomerase signaling
tRNA splicing signaling
